# An Observational Study of the Implementation of the Tobacco-Free Film and Television Policy in India

**DOI:** 10.1177/1179173X231205377

**Published:** 2023-10-19

**Authors:** Nalin Singh Negi, Vineet Munish Gill, Meena Maharjan, Praveen Sinha, Pallavi Puri, Vaishakhi Mallik, Sandra Mullin, Fikru Tullu, Nandita Murukutla

**Affiliations:** 1 525891 Vital Strategies, New Delhi, India; 2 93774 World Health Organization, New Delhi, India; 3 Vital Strategies, New York, NY, USA

**Keywords:** tobacco-free film, anti-tobacco, mass media campaigns, policy, static message, disclaimer, India, film rule

## Abstract

**Background:**

Positive portrayals of tobacco use in entertainment media can normalize and perpetuate use. In 2012, the Government of India implemented the Tobacco-Free Film and Television Rules, a first-of-its-kind comprehensive regulation to restrict tobacco depiction in films and television programs. Two complementary studies were undertaken to assess the implementation of the film rules on television and in movie theaters.

**Methods:**

In the first part, movie theater observations and exit surveys were conducted from Feb. 3 to March 24, 2015. In total, 308 movie theaters were selected for the observation of films. A total of 3080 exit surveys were conducted to assess moviegoers’ reactions toward the film rule. The second part comprised the systematic observation of 424 prerecorded television programs that aired from Nov. 20 to Dec. 30, 2015.

**Results:**

Compliance with the Tobacco-Free Film and Television Rules policy was lower on television than in movie theaters. While 66% of television programs with tobacco scenes implemented at least 1 of the 3 elements of the film rule, not a single program executed all required elements correctly. In movie theaters, 99% of films that contained tobacco scenes implemented at least one element of the film rule. However, all elements of the film rules were implemented correctly during 27% of the films observed. Exit surveys showed that among moviegoers who recalled viewing at least one element of the film rule, there was increased concern about tobacco’s harms and intentions to quit.

**Conclusion:**

Implementation of the film rules was higher in movie theaters than on television, though there were gaps in implementation for both. Despite inconsistent application, audience reactions to the anti-tobacco messages were favorable, with increased concern about tobacco’s harms and intention to quit. Overall, the film rules offer a strong tool for countering tobacco promotion, reaching hundreds of millions with anti-tobacco messaging.

## Introduction

Tobacco use is the single most preventable cause of premature death and disease, claiming more than 8 million lives worldwide each year.^1^ India is home to the second largest number of tobacco consumers in the world, with an estimated 1.35 million deaths annually attributed to tobacco use.^2^ A large body of evidence has shown that positive portrayals of tobacco use in media and entertainment can normalize and perpetuate use.^3-6^ India has the world’s second largest population and one of the largest film and television industries, so Indian media and entertainment wields significant influence over millions of people and their tobacco use behaviors, especially youth, who are particularly susceptible to social and environmental influences to use tobacco.^7,8^ India is the world’s largest producer of films, producing more than 1500 films each year with a footfall of more than 2.1 billion in cinemas worldwide.^9^ Films are also frequently broadcast on television in India, alongside a multitude of other general entertainment shows that are programmed to entertain viewers, who watch an average of three hours and 42 minutes of television a day.^10^ According to the Broadcast Audience Research Council, the number of Indian households that own a television set continues to grow, with approximately two-thirds of all households now owning a set (210 million households).^11^ Research has shown that tobacco media advertising^12^ and exposure to cigarette brand names or visuals of actors smoking on television and in films is related to increased smoking among Indian youth.^13,14^ Despite this, a recent analysis of the top ten grossing Bollywood movies from 1996 to 2013 found an average occurrence of 4 tobacco scenes per film.^15^

Policies that ban tobacco advertising, promotion, and sponsorship (TAPS), including in entertainment content, are effective in preventing or reducing tobacco consumption.^13,16,17^ Such policies have a large population impact, thereby reducing the initiation and continuation of tobacco use. Hence, they are considered to be a tobacco control “Best Buy.”^18^ Moreover, a large body of evidence, including from India, has found that anti-tobacco mass media campaigns can denormalize tobacco use, prevent its uptake and reduce consumption.^13,16,17,19,20^ Therefore, banning TAPS and conducting anti-tobacco mass media campaigns are integral to policy efforts to reduce tobacco consumption. They constitute core interventions in the World Health Organization’s MPOWER policy package for tobacco control, and provisions within the Framework Convention on Tobacco Control.

Nearly one-fifth of the world, including India, was covered by a “highest level” TAPS ban in 2018.^1^ However, entertainment and emerging media poses significant challenges to these bans. For example, while these policies often ban the tobacco industry from sponsoring tobacco content in films, they do not often include non-sponsored depictions of tobacco.^21^ This underscores the need for new TAPS policies to respond to evolving media and tobacco advertising landscapes.

### The Tobacco-Free and Television Rules Under the Cigarettes and Other Tobacco Products Act (COTPA)

To address the growing burden of tobacco, in 2003 the Government of India passed a comprehensive tobacco law known as the Cigarettes and Other Tobacco Products Act (COTPA). The law prohibits the advertisement and display of products in multiple ways. Section 5 of the COTPA amendments (Rules issued in 2011, 2012), commonly referred to as the “film rules,” came into effect on Oct. 2, 2012 (see Appendix 1). The film rules require that all films and television programs produced in India, or produced elsewhere but aired in India, that depict tobacco products or tobacco use, must meet the following requirements:

- Strong editorial justification to the Central Board of Film Certification on the necessity of the display of tobacco products
- Government approved anti-tobacco health spots (public service announcement) of a minimum of 30 seconds duration at the beginning and during the middle of a film/TV program.
- An audio-visual disclaimer on ill effects of tobacco use of a minimum of 20 seconds, at the beginning and middle of the film/TV program
- A static health warning message to appear onscreen each time the tobacco product is depicted. There are additional specific requirements for this warning that are described in Appendix 1.

For content produced before Oct. 2, 2012, the act requires that the anti-tobacco health spots and the static health warning accompany the content wherever applicable. These provisions are among the most comprehensive regulations globally pertaining to regulation of TAPS in entertainment media. The rules not only seek to curtail TAPS, but also, because the cost of implementing the film rules is borne by the content distributors and producers, they provide the Government of India with a cost-efficient means of protecting the public from tobacco promotion.

The Government of India’s film rules offer a global first in countering TAPS and helping to denormalize tobacco use through the use of strong anti-tobacco messaging integrated into entertainment content. The film rule has the ability to reach not only the millions of consumers of India’s entertainment content within its borders but also those in other countries who consume this content through satellite networks. In an age where cross-border TAPS is of increasing concern, film rules offer a potential deterrent to such practices. In addition, experience from India shows that after tobacco advertising was banned on traditional channels in 2004, there was a marked increase of tobacco imagery in films as the tobacco industry looked for alternative ways to promote products.^22^ Research demonstrates that after the film rule was introduced, there was a reduction in tobacco depictions,^23,24^ particularly in youth-rated movies. Thus, film rules can help to address loopholes in TAPS regulations and deter filmmakers from depicting tobacco use or brands in their films.

In 2015, Vital Strategies, a global public health organization, in partnership with the World Health Organization (WHO) and under the guidance of the Government of India, undertook an evaluation study to monitor the implementation of the film rules in movie theaters nationally and on television. The Government of India released key highlights from these studies in February 2017 in a consultative workshop in Mumbai, India. Participants included representatives from the Ministry of Health and Family Welfare and Ministry of Information and Broadcasting and representatives of the film producer’s guild, as well as WHO and civil society organizations. This paper builds on the highlights released previously and provides a more thorough description of the study findings.^25^

It also helps to address several gaps in the literature: To date, some studies have assessed the implementation of the Tobacco-Free Film and Television Rules in films shown in movie theaters,^26^ but no studies have assessed implementation on television. Evaluating compliance with the film rules on television is a priority, given that television viewership is large and growing.^27^ Television programming is consumed repeatedly and frequently and is less regulated in terms of age restrictions than movies shown in theaters, offering ample opportunity for audiences—and particularly younger audiences—to be exposed to tobacco. In addition, previous studies on the implementation of the film rules were limited to films in particular regions and languages screened in movie theaters.^26^ This study aims to provide a comprehensive picture of policy implementation in movie theaters nationwide and on television, and to gauge moviegoer recall of the elements of the film rules, their reactions toward them and whether there was any change in their knowledge and attitudes toward tobacco as a result of having been exposed to them.

## Methodology

To assess the implementation of the film rules in movie theaters and on television, 2 complementary studies were conducted. One involved observations of films in national theaters and exit surveys with moviegoers and the other consisted of observations of pre-recorded television programs. For both studies, all observers spoke the language of the content they were observing. The methods for each of these studies are described separately below, but the results of the studies are presented together to better aid interpretation of the findings.

### Study 1: Movie Theater Observation and Exit Surveys

#### Film Selection

To help ensure a systematic and representative selection of films, a multistage selection procedure was implemented. Across each of the 4 regions of India, one metropolitan city, 2 tier 1 cities and two smaller cities were randomly selected. Movie theaters were then selected at random within each city using a list obtained from the Ministry of Information and Broadcasting. In total, 308 movie theaters were selected for film observation: 73 in the north, 81 in the east, 79 in the west and 75 in the south; 167 from a metropolitan city, 100 from tier 1 cities and 41 from smaller cities (see Appendix 2, Table 2). In each theater, a single film was randomly chosen for observation; a quota was set to ensure that 50% of these contained a tobacco scene (the determination was made a priori through anecdotal knowledge of the films and was then confirmed through observation). Some films were included multiple times in different theaters; a total of 74 unique films were observed across the 308 movie theaters.

#### Observation Questionnaire and Procedure

An observation questionnaire was designed to record each instance of tobacco depiction in films in selected movie theaters and whether and to what extent film rule elements had been implemented (see Appendix 3). The observation questionnaire was divided into 2 sections: The first section contained general information about the film, such as the genre, rating and language and the second section assessed the total number of scenes in the film where tobacco was consumed and the implementation of the 3 observable elements of the film rule, including the presence or absence of each element of the film rule and the extent to which each element was compliant with the specifications of the regulation.

#### Exit Surveys with Moviegoers

The movie theater observations and exit surveys were conducted from Feb. 3 to March 24, 2015. Observer/interviewers observed and coded films in movie theaters according to the questionnaire. They later surveyed adults aged 15 to 50 who had just completed watching the same film. The trained observers/interviewers positioned themselves outside the theater to conduct the survey. The purpose of these surveys was to gauge moviegoers’ knowledge and attitudes toward tobacco, whether they recalled seeing elements of the film rule and their reactions to the elements, if they recalled seeing them. Prior to being rolled out, the questionnaire was piloted with a small sample of the target population.

To ensure that participants were selected systematically, the observer/interviewer solicited participation from every 10th person. They then completed an in-person survey using a structured questionnaire after the participant’s eligibility for the study was established and their written consent was obtained. The surveys were conducted in local languages and lasted for an average of 20 minutes. In each of the selected movie theaters, 10 exit surveys were conducted, resulting in a final sample size of 3080.

#### Sample Size Estimation

To estimate the sample size, we used the following equation. The sample needed to be representative of the entire country; capture zonal differences; and have appropriate statistical significance.

$$S=\frac{Z_{1-\alpha }^{2}\times P(1-P)\times D_{eff}}{S_{e}^{2}}$$

S Sample Size

Z_1-α_ Z-value for confidence interval (taken as 95%)

P Proportion of the key characteristic (taken as 50% for maximum heterogeneity)

D_eff_ Design Effect (taken as 1.25 for multi-stage sampling)

S_e_ Standard error (taken as 4%)

The minimum sample size required with this calculation was found to be 750 for each of the 4 zones of India. Thus, a total sample size of 3000 was deemed necessary for the study.

#### Questionnaire

An evidence-informed structured questionnaire (see Appendix 4) was developed to measure the following:

*Sociodemographic characteristics* including gender, age, education level, socioeconomic status, current tobacco use, frequency of tobacco use and history of tobacco use.

*Knowledge and attitudes about tobacco consumption,* which were assessed by asking all exit survey participants to rate statements on a five-point Likert scale (1 = strongly disagree, 5 = strongly agree). Statements included: “people important to me believe that I should not use tobacco”; “tobacco cause serious illness”; “exposure to smoke from another person’s cigarette (or passive smoking) causes serious illnesses in nonsmokers”; and “the law that prohibits smoking in indoor places will benefit public health.”

*Recall of the film rule and reactions generated,* which was assessed by asking participants if they saw the anti-tobacco health spots, audio-visual disclaimer and/or static health warning before or during the film they just watched. Following the recall question for each element of the film rule, participants who had recalled at least one element of the film rules were asked to rate statements about the element on a five-point Likert scale including: “it was easy to understand”; “it made me concerned about the health effects of tobacco”; and “it made me more likely to quit tobacco” (See Table 6).

### Study 2: Observation of Television Programming

#### Content Selection

Television content was systematically selected and pre-recorded for coding by trained researchers to assess the implementation of the film rules. Five weeks’ worth of content was selected for coding (Nov. 20 to Dec. 30, 2015). In this paper, by “television” we mean a device on which one watches various programs and films through cable or direct-to-home subscriptions.

To ensure systematic and representative selection, the content was selected through a multistage random selection procedure. In the first stage, channels were selected systematically, thereafter, content for coding was selected systematically from within each channel. To select channels for the study, the universe of 446 television channels available in India according to the Television Audience Measurement (TAM 2015),^28^ were categorized by ownership (international, national and regional), and within each category, they were sorted by media reach (according to TAM) into 3 categories (high, medium and low reach). From within each category, 5 channels were selected at random, resulting in a total of 45 channels (see Appendix 2, Table 1). To select the content for coding, a minimum of 9 hours of programming was recorded from each channel during the study period, with one-third of the content from prime-time hours (prime time is defined as 8 pm to 11 pm) and two-thirds of the content from non-prime time hours. In total, 413 hours and 27 minutes were observed and coded, corresponding to 424 full programs.

**Table 1.** Characteristics of Movies With and Without Tobacco Depictions.

Variables	Movie status						
	Total	Included scenes with tobacco content	Did not include scenes with tobacco content	95% Confidence interval			
	N = 308	n = 153	n = 155				
	%	%	%	OR	LL	UL	*P*-value^a^
Genre of the movie							<.001*
Action	33	38	62	Ref			––
Adventure	8	33	67	.808	.32	2.06	.655
Comedy	14	62	38	2.63	1.25	5.50	.011*
Romantic	21	71	29	4.00	2.05	7.78	<.001*
Thriller	18	43	57	.60	2.00	.61	.597
Others	7	50	50	.33	1.62	.62	.329
Central board of film certification rating							.392
Universal (U)	13	39	62	Ref			––
Universal with an adult theme (U/A)	74	51	49	1.64	.82	3.29	.162
Adult (A)	13	55	45	1.96	.80	4.794	.143
Language							.001
Hindi	71	44	56	Ref			––
Telugu	10	81	19	5.34	2.11	13.53	<.001*
Kannada	6	65	35	2.35	.84	6.58	.104
Bengali	5	71	29	3.20	.98	10.53	.055
Others	9	41	59	.88	.39	1.99	.076

Abbreviations: OR, odds ratio; ref, reference category; LL, lower limit; UL, upper limit.

*Significant at *P*-value less than .05.

^a^Results are based on univariable logistic regression.

#### Observation Questionnaire and Procedure

An observation questionnaire was designed to record each instance of tobacco depiction (see Appendix 5). The questionnaire was divided into 4 parts: the first included channel and show information; the second, details on the presence or absence and extent of each observable element of the film rule, and whether or not there were any tobacco scenes in the show or film (yes/no) and if so, what type of tobacco product was observed^1^; the third examined whether tobacco/pan masala branding was present; and the fourth asked whether there were any other public health advertisements shown. Six researchers were trained to identify the government-approved health warnings under the film rule, which included: the 2 preapproved anti-tobacco health spots known as “Child” (a spot about the harms of secondhand smoke exposure) and “Dhuan” (a spot supportive of the enforcement of the smoking ban in public places); an audio-visual disclaimer and the static health warning (see Appendix 6). All 6 researchers first coded the same 10 shows to establish inter-rater reliability (IRR = .9). The researchers then proceeded to code the remaining content independently and separately.

### Analysis

Data were analyzed using the statistical analytical package IBM SPSS 25 version. Descriptive analysis was performed to provide descriptive summaries of the sample and measures, including percentages for categorical variables and means and standard deviations for continuous measures. Fisher’s exact (Freeman-Halton) test, an alternative to the Pearson chi-square test,^29^ was used to assess the association between groups among categorical variables with 2 or more levels. Univariable binary logistic regression was used to identify whether a relationship exists between a dependent variable (content with/without tobacco scenes), with content without tobacco scenes as the reference category and categorical independent variable, generating a crude odds ratio. For movie and television channel genre, action films and entertainment channels, were respectively chosen as references because they were presumed to have higher quantities of tobacco scenes than other genres. Likewise, multivariable logistic regression was performed to measure the effect of the film rule elements on the outcome measures (knowledge and attitudes toward tobacco consumption),” adjusting for the covariates: gender, age, education level, socioeconomic status, and status of tobacco use. The outcome variables were dichotomized where “1” represented “somewhat agree, strongly agree” and “0” represented “strongly disagree, somewhat disagree, neither agree nor disagree.”

## Results

### Observational Studies: Characteristics of Films in Theaters

Table 1 presents the characteristics of films in theaters that were observed for this study and whether or not they had scenes with tobacco. It was approximately 2.6 times more likely that comedy films had tobacco depictions (*P = .011*) than action films, and 4 times more likely for romance films to have tobacco depictions (*P < .001*) than action films.

**Figure 1.**
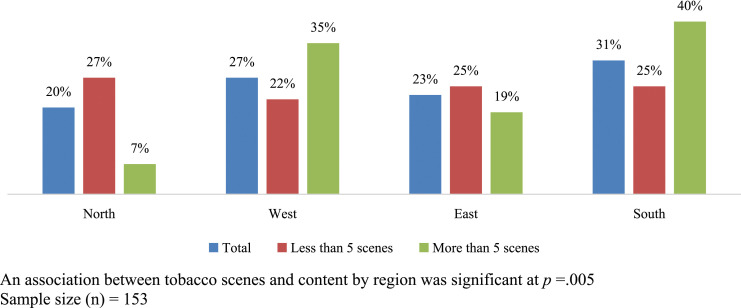
Movies with tobacco scene content by region.

Films in regional languages were more likely to contain tobacco content than films in Hindi: Films in Telegu were approximately 5.3 times more likely to contain tobacco scenes *(P < .001).* Tobacco was most frequently depicted in films shown in theaters in the southern (40%) and western (35%) regions of the country (Figure 1).

### Observational Studies: Characteristics of Television Programming

Table 2 presents the characteristics of the television programs that were observed for this study and whether or not they had tobacco content. Out of 424 randomly selected television programs (some of which were movies shown on TV), tobacco was depicted in nearly a quarter (22%). Tobacco was more likely to be featured in content on movie channels (OR = 28.9) and music channels (OR = 7.4), than entertainment channels. Nearly three-quarters of content that was rated “U” (all ages) had tobacco scenes (72%), while more than three-quarters of “U/A” programs (parental guidance for children under 12) had tobacco scenes (84%) (OR = 1.98, *P = .286*) (Table 2).

**Table 2.** Characteristics of Television Programming With and Without Tobacco Depictions.

	Movie/show status						
	Total	Included scenes with tobacco content	Did not include scenes with tobacco content				
	(N = 424)	(n = 94)	(n = 330)	95% CI			
Variables	%	%	%	OR	LL	UL	*P*-value^a^
Channel ownership							.464
National	30	22	78	Ref			__
International	37	20	80	.89	.50	1.58	.682
Regional	33	26	75	1.24	.70	2.20	.452
Channel genre							<.001*
Entertainment	54	11	89	Ref			__
Movies	11	79	21	28.9	12.9	64.9	<.001*
Music	9	49	51	7.40	3.45	15.87	<.001*
Kids	3	0	100	.00	.00	–	.999
Others	23	13	87	1.19	.59	2.43	.625
Central board of film certification rating							.286
Universal (U)	48	72	28	Ref			__
Universal with an adult theme (U/A)	52	84	16	1.98	.56	6.96	
Language							.393
English	35	17	83	Ref			__
Hindi	32	24	76	.66	.37	1.18	.162
Malayalam	10	30	70	1.37	.62	2.99	.436
Tamil	8	26	74	1.10	.47	2.60	.822
Others	16	24	76	1.00	.50	1.99	1.000
Timing of show/movie (total as per defined quotas)							.076
Prime time	36	18	82	Ref			__
Non-prime time	64	25	75	1.58	.95	2.62	
Date of production							<.001*
“Old” show/movie (prior to 2012)	14	75	25	Ref			__
“New” show/movie (after 2012)	86	14	86	.05	.03	.10	

Abbreviations: OR, odds ratio; ref, reference category; LL, lower limit; UL, upper limit.

*Significant at *P*-value less than .05.

^a^Results are based on univariable logistic regression.

**Figure 2.**
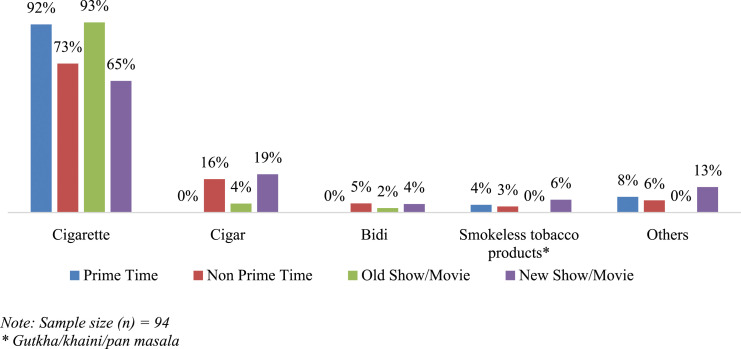
Depiction of tobacco products on television by airtime and production year.

It was 5% more likely for tobacco to be depicted in newer shows or movies shown on TV (after 2012) than in older TV shows or movies (prior to 2012) (see Table 2). Cigarettes were consistently the most frequently depicted tobacco product, however, there was a reduction in the portrayal of cigarettes in newer TV movies and shows (65%) when compared to older TV movies and shows (93%) (see Figure 2). Notably, there was an increase in the portrayal of other products: cigars (+375%) and smokeless tobacco products (+600%).

### Observed Implementation of the Film Rules: Movie Theaters

Nearly all of the films that contained tobacco scenes implemented at least one of the 3 elements of the film rule (99%) (see Table 3). However, all key elements of the film rules were implemented fully and correctly in only 27% of those films. The most common lapse in implementation was the display of the static health warning, which was correctly shown in only 30% of films. In 71% of the films with tobacco scenes, both anti-tobacco health spots were fully and properly shown—in the beginning and middle of the films and in the same language as that of the film. Notably, 56% of films without tobacco scenes employed at least one of the film rule elements in any form, namely the audio-visual disclaimer (52%).

**Table 3.** Implementation of the Film Rules on Television and in Movie Theaters.

Film rule elements	On TV	Movie Theaters		
	Content with tobacco scenes	All Movies	Movies with tobacco scenes	Movies without tobacco scenes
	n = 94	N = 308	n = 153	n = 155
	%	%	%	%
Presence of film rule elements (PSA, disclaimer, static message)				
All elements shown correctly	0	13	27	0
At least 2 elements shown correctly	0	27	61	19
At least one element shown correctly	3	31	94	49
No elements shown correctly	97	29	6	50
Element shown in any form	66	77	99	56
Government approved public service announcements (PSAs)				
Both PSAs shown correctly^a^	2	46	71	21
Included one at the start and one in the middle correctly	5	56	86	25
Included at least one either at the start or in the middle correctly	14	67	94	39
PSA shown in any form	14	69	97	41
Disclaimer				
Disclaimer shown correctly^b^	1	60	74	47
Disclaimer shown in any form	1	65	77	52
Static message				
Static message shown correctly^c^	0	15	30	0
Static message shown in any form	65	43	86	0

*all the elements a, b, and c mentioned below are shown correctly on the film or television shows/movies.

^a^both anti-tobacco health spots or messages shown at the beginning and the middle of the film or the television shows and movies.

^b^disclaimer shown in the same language as the film or television shows/movies.

^c^static message shown in the same language, with a font in black color on a white background and placed at the bottom of the screen.

### Exit Surveys in Movie Theaters

#### Sociodemographic Characteristics of Moviegoers

The sociodemographic characteristics of participants in the exit surveys are presented in Table 4. There were statistically significant differences in the characteristics of participants who had just exited from films that contained tobacco scenes compared with those who had just exited from films without tobacco scenes. Specifically, those who had just exited from a film with a tobacco scene were more likely to be women, younger, have a university degree, and be in the southern region of the country. They were also less likely to report that they were current tobacco users.

**Table 4.** Characteristics of Participants in Exit Surveys.

	Total	Movies with tobacco scenes	Movies without tobacco scenes	*P*-value^a^
	N = 3080	n = 1514	n = 1566	
	%	%	%	
Women	44	53	37	<.001*
Age (years)				<.001*
Less than 29	43	45	55	
Above 29	43	55	45	
Education				<.001*
Secondary or less	17	14	21	
High school degree	24	25	25	
College/university degree	46	49	40	
Post-graduate degree	13	12	14	
Zones				<.001*
North	24	19	30	
West	27	27	27	
East	25	23	25	
South	24	31	18	
Socio-economic class (SEC)				.135
SEC A	43	45	42	
SEC B	37	36	38	
SEC C	18	18	18	
SEC D	2	1	2	
**Tobacco user (yes)**	22	19	26	<.001*
**Likely to quit using tobacco (definitely/very likely)**	28	31	26	.078

^a^Results are based on Fisher’s exact test.

^*^Significant at *P*-value less than .05.

#### Moviegoer Recall of the Film Rule

In films with tobacco scenes, 93% of participants recalled seeing some form of anti-tobacco advertisement and 69% to 79% of participants specifically recalled seeing one of the government’s 3 anti-tobacco film rule elements (see Table 5). Notably, 43% of people who had just exited from films without tobacco scenes recalled seeing a form of anti-tobacco advertisement, with 37% to 42% of people specifically recalling one of the film rules elements. Of the different film rule elements, the anti-tobacco health spots were most effective in making people concerned about tobacco use (73%), while the static health messages were least effective (6%).

**Table 5.** Participants’ Recall of Public Health Advertisements and Film Rules Elements.

		Movies with tobacco scenes	Movies without tobacco scenes	*P*-value^a^
	N = 3080	n = 1514	N = 1566	
	%	%	%	
Recall of any advertisements				
Anti-tobacco	68	93	43	<.001*
Anti-alcohol	31	45	18	<.001*
Cleanliness	19	21	18	.051
Vaccination	7	8	6	.001*
Recall of government's anti-tobacco messaging				
Anti-tobacco PSA 1 (Dhuan)	54	70	39	<.001*
Anti-tobacco PSA 2 (child)	60	79	42	<.001*
Disclaimer	60	79	41	<.001*
Static message	53	69	37	<.001*
The type of message channel that made people most concerned about tobacco use				<.001*
Advertisements	73	73	72	
Disclaimer	20	21	20	
Static message	6	6	5	
Noticed any of the characters in the movie: (Yes)				
Using tobacco (smoking/chewing)	54	89	21	<.001*
Consuming alcohol	73	82	64	<.001*

^a^Results are based on Fisher’s exact test.

*Significant at *P*-value less than .05.

#### Moviegoer Reactions Toward the Film Rule

Reactions toward elements of the film rule are presented below (Table 6). The vast majority of participants who recalled the elements of the film rule described the elements as easy to understand (96%) and indicated that the elements made them stop and think (88%), increased their concern about tobacco’s health harms (91%) and increased the likelihood that they would talk about the message (90%). Approximately 3 in 4 people who used tobacco said that the element(s) made them more likely to quit (76%).

**Table 6.** Reactions of Moviegoers Toward the Film Rule Elements (% Who Strongly Agree/Somewhat Agree).

	PSA 1 (n = 1670)	PSA 2 (n = 1845)	Disclaimer (n = 1833)	Static message (n = 1635)	Average rating
It was easy to understand	95	96	96	96	96
It made me stop and think	88	89	88	87	88
It made me concerned about the health effects of tobacco	91	90	91	91	91
I am likely to talk about this advertisement to other people	91	90	90	90	90
It made me more likely to quit tobacco	77	74	75	77	76

### Association Between Recall of Film Rule Elements and Knowledge and Attitudes Toward Tobacco

Table 7 describes the associations between recall of any element of the film rule and knowledge and attitudes toward tobacco. There were no statistically significant differences in knowledge or attitudes toward tobacco as a result of participants having recently seen the film rule implemented.

**Table 7.** Knowledge and Attitudes Among Participants Aware and Unaware of the Film Rule Elements.

		Movie with tobacco scenes					Movies without tobacco scenes				
		Unaware^a^	Aware^b^	Adjusted	Confidence interval		Unaware^a^	Aware^b^	Adjusted	Confidence interval	
Statements (% agree)	N = 3080	n = 84 (%)	n = 1430 (%)	OR^	LL	UL	n = 663	n = 903 (%)	OR^	LL	UL
People important believe me that I shouldn't use tobacco	94%	99	95	.00	.00	-	96%*	91	.96	.48	1.92
Smoking causes serious illness	94%	94	97	1.35	.31	5.97	94%*	90	.56	.21	1.51
Exposure to smoke from another person’s cigarette (or passive smoking) causes serious illnesses in non-smokers	94%	99	96	.00	.00	-	92%	91	1.37	.65	2.88
The law that prohibits smoking indoor places will benefit the public health	93%	94	95	1.23	.41	3.70	93%*	90	.73	.37	1.45

^a^Unaware of any element of the film rule.

^b^Aware of any element of the film rule.

Abbreviation: OR, odds ratio; LL, lower limit; UL, upper limit.

^Covariates adjusted for include gender, age, education, tobacco users.

*Significant at *P*-value less than .05.

### Observed Implementation of the Film Rules: Television

Overall, implementation of the film rule on television was low (see Table 3). While 66% of programs employed at least one element of the film rule in some form—and 3% did so correctly—none of the programs with tobacco depictions implemented all 3 elements of the film rule as required. Approximately 14% of television programs employed the government-approved anti-tobacco health spots, but only 2% carried the spots correctly at the beginning and in the middle of the programs. The audio-visual disclaimer was implemented least often—shown only in one instance. In this instance, the disclaimer was shown in the same language of the program as per the film rule, but it was only shown before the program and not in the middle of the program as well. In addition, the disclaimer was not played completely and it was broadcast in low resolution rendering images of poor quality. The static health warning was the most frequently implemented element of the film rule, observed in 65% of programs with tobacco. However, it was not once properly implemented, that is, shown in the same language as the film, with a black font on a white background and placed at the bottom of the screen.

## Discussion

The insights that our study offers are 2-fold. First, we offer a first look into how the film rules are being implemented on television, as well as in movie theaters nationwide. These findings and our interpretations in the context of India’s experience with industry pushback, may be instructive to other countries looking to implement similar regulations. Second, we uncover variations in the volume and frequency of tobacco depictions in entertainment content by movie genre/TV channel type, production date, type of tobacco product depicted and language.

Our findings demonstrate that overall compliance with the film rule is low and is significantly lower on television than in movie theaters. While all 3 components of the film rule were properly implemented in 27% of films with tobacco imagery in movie theaters, this was not the case for a single program or film on television. At least one component of the film rule was implemented in some form in 99% of films with tobacco imagery in movie theaters compared with 66% of programs on television. Several implementation gaps were observed in both movie theaters and on television. The anti-tobacco health spots and audio-visual disclaimer were shown either before the movie or during intermission, but not both as required, were not those approved by the Ministry of Health, and/or were broadcast in low resolution. The static health warnings were frequently not in the same language as the film or program; used language that was entirely different from what was indicated in the regulation; used font that was small or in the wrong color; or the background color or placement of the text was off—all contributing to making it more difficult for viewers to understand the messages.

These findings suggest that key stakeholders, including film distributors and producers, and in particular, television broadcasters, are not fully complying with the policy. In fact, the entertainment industry’s opposition to the regulation has been well documented,^30-32^ and penalties for noncompliance have been inconsistent.^24,30^ However, lower levels of compliance may also stem from confusion in interpreting and applying all of the film rule elements and call attention to a need for efforts to improve understanding of the rules among stakeholders.^24^

When the film rules were in the development stage, television broadcasters were concerned that anti-tobacco health spots would consume advertising time and cause people to change channels.^30^ This might account in part for the lower use of the anti-tobacco health spots on television (14% on television compared to 97% in films). Filmmakers, on the other hand, were most resistant to the static health warning messages.^30,32-34^ Our study demonstrates that despite this resistance, static health warnings were the most frequently implemented element on television, and the second most frequently implemented element in movie theaters. However, the static health warnings were not intended to reach consumers at all; they were designed as an “upstream solution” intended to deter filmmakers from including tobacco in films during the production process.^35^ Upstream solutions help to reduce tobacco content at the source, putting the onus on those who create content rather than those who consume it.^35^

Our findings show that moviegoers’ reactions to the film rule elements were highly positive, and that the health messaging successfully increased concern about the harms of tobacco and made 3 in 4 people who use tobacco say they’d be more likely to quit. The anti-tobacco health spots were the most effective element in making people concerned about tobacco use. More moviegoers recalled the messaging in the spot “Child,” which focuses on the negative effects of secondhand smoke on families, than “Dhuan,” which focuses on the enforcement of smoke-free laws public places. Research shows that the most effective type of anti-tobacco messaging tends to be personal, highly emotional stories focused on the negative health effects of tobacco, which was the style of messaging employed by “Child.”^36,37^ Future public service announcements that are put into circulation through the film rules may opt for a similar message style.

However, not only do the effectiveness of health spots depend on the type of message, but also on the intensity and duration of the message.^37,38^ Sustainable changes to attitudes and behavior do not occur immediately, but are the result of multiple exposures to a health message.^39,40^ The film rule policy offers the frequent and widespread exposure to anti-tobacco campaign messaging that has been found to be important for reducing population tobacco use and securing ongoing behavior change.^40^ However, a limitation of this study is that none of the questions asked in the exit surveys with moviegoers addressed message exposure. As a result, we did not gather a sense of how often people had seen the public service announcements prior to that day; at that point the PSAs had been in theaters for almost a year and had been in circulation in India for 8 years. This may explain why we found no statistically significant differences in knowledge and attitudes about tobacco as a result of participants having recently seen the film rule implemented. Another possible explanation for the lack of statistical significance is social desirability bias in answers to questions about the harms of tobacco. This is a risk in survey research on tobacco, especially as tobacco use is increasingly seen as non-normative.^41^

Our findings also reveal variations in the volume and frequency of tobacco depictions in entertainment content by movie genre and channel type, type of tobacco product depicted, region and language. Overall, we found that approximately one-quarter of television programs had tobacco content. This is on par with what other studies have found on television in New Zealand,^42,43^ but is less than what was found in the U.K., where one-third of TV programs had tobacco content.^44^ In our study, tobacco imagery was more likely to be observed on movie and music channels than entertainment channels. We also found that it was 5% more likely for tobacco to be depicted in newer shows or movies shown on TV (after 2012) compared to in older TV shows/movies (prior to 2012). This is inconsistent with findings that show a significant reduction of tobacco content in Bollywood films after the film rules were introduced.^23^ This may represent a limitation of our study: when selecting television shows, we did not identify whether episodes were older episodes that were being rerun and were thus categorized as “new” when they were in fact originally released prior to 2012. However, it is also possible that despite declines in tobacco scenes in Hindi-language films, there was an increase in content in regional languages or in television shows; future research may investigate whether this is the case. Further, as this study was conducted in 2015, it did not cover online video streaming, also known as over-the-top services like Hotstar, Netflix, Zee5, Amazon Prime, Hulu, Disney and others, which gained popularity after 2015. Studies from the United Kingdom and United States have suggested that content on these services have more tobacco imagery than traditional television, and a study from India found that tobacco imagery was common in streaming series popular among young people, and were higher in foreign productions.^45-47^ In 2023, the Government of India extended the film rules to over-the-top platforms.^48^

Cigarettes were consistently the product that was most frequently shown on television. However, in newer programs/movies there was a reduction in the portrayal of cigarettes and an increase in the portrayal of other products, namely cigars and smokeless tobacco products. In movie theaters, films in regional languages were more likely to depict tobacco than films in Hindi, which is consistent with findings from previous studies.^26^ Films in Telegu were 5 times more likely to contain tobacco scenes than films in Hindi. In movie theaters, romance and comedy films were more likely to contain tobacco scenes than action films. Historically, smoking in Indian cinema has been tied to “romance, style, tragedy and rebellion,”^7^ as well as masculinity and power and thus is commonly depicted in romance films. The finding that comedy films are a genre that is particularly likely to depict tobacco may be worthy of additional research attention, as this is a popular genre in India.^49^ Studies from the U.S. and U.K. have also found that tobacco depiction is prevalent in comedy entertainment content.^50,51^

Earlier drafts of the film rules had required that movies and television programs with tobacco scenes automatically be rated “A” for adults ages 18 or above (2006 draft) and “U/A” parental guidance for youth ages 12 and under (2011 draft).^30^ However, due to resistance from the film industry, these rating requirements were removed in the final regulation,^26^ which means that youth are continuing to be exposed to tobacco use in entertainment content. Consistent with earlier studies,^14,26^ we found that this content continues to be accessible to young people: some films in theaters and a substantial amount of content on television—more than 4 out of every 10 programs with tobacco depictions—are still receiving a “U” rating deeming them appropriate for all ages. In addition, 18% of content with tobacco scenes on television are aired during prime time, when there is high youth viewership.^52^ Time and time again, studies have shown that onscreen tobacco use can influence youth to start smoking.^14,53-55^ Therefore, further steps need to be taken to ensure that youth are protected from viewing tobacco on screen.

## Conclusion

This study has shown that the film rules are not being fully implemented in accordance with the requirements laid out in Section 5 of COTPA. While compliance with the rules is far better for films shown in movie theaters than for movies/programs aired on television, there are gaps in implementation across both in terms of the frequency, quality and accuracy of the health messages being shown. Despite inconsistent implementation of the film rules, audience reactions to the anti-tobacco messages in films are favorable and indicate an increased concern about tobacco’s harms and self-reported intention to quit among those who use tobacco. Overall, the Government of India’s film rules offer a strong tool for countering TAPS, in particular the positive portrayals of tobacco use in films and television programming that can normalize its use and influence youth. Through these rules, hundreds of millions of people both within India and abroad are reached with anti-tobacco messaging, and with improved implementation, reach and effectiveness will only increase.

## Supplemental Material

Supplemental Material - An Observational Study of the Implementation of the Tobacco-Free Film and Television Policy in India Click here for additional data file. Supplemental Material for An Observational Study of the Implementation of the Tobacco-Free Film and Television Policy in India by Nalin Singh Negi, Vineet Munish Gill, Meena Maharjan, Praveen Sinha, Pallavi Puri, Vaishakhi Mallik, Sandra Mullin, Fikru Tullu, and Nandita Murukutla in Tobacco Use Insights

## References

[1] World Health Organization . WHO report on the global tobacco epidemic, 2019: Offer Help to Quit Tobacco Use. 2019.

[2] Tata Institute of Social Sciences (TISS) , Mumbai and ministry of health and family welfare, government of India. Global Adult Tobacco Survey 2, 2016-2017.

[3] Sargent JD . Smoking in movies: Impact on adolescent smoking. Adolesc Med Clin. 2005;16(2):345-370. 16111622 10.1016/j.admecli.2005.02.003

[4] Sargent JD Hanewinkel R , Impact of media, movies and TV on tobacco use in the youth. The Tobacco Epidemic. 2015;42:171-180.

[5] National Cancer Institute . The role of the media in promoting and reducing tobacco use. In: US Department of Health and Human Services. National Institutes of Health; 2008.

[6] Surgeon General U . Preventing Tobacco Use Among Youth and Young Adults. A Report from the Surgeon General. US Dep Heal Hum Serv; 2012:1395.

[7] World Health Organization . Bollywood: Victim or Ally. A Study on the Portrayal of Tobacco in Indian Cinema. World Health Organization; 2003.

[8] Albornoz LA . Diversity and the Film Industry: An Analysis of the 2014 UIS Survey on Feature Film Statistics. UNESCO Institute for statistics; 2016.

[9] Jain N Soneja T Ahluwalia J . Indywood: The Indian Film Industry. Deloitte; 2016.

[10] Broadcast Audience Research Council (BARC) India . What India Watched 2019. BARC yearbook; 2019.

[11] Broadcast Audience Research Council (BARC) India . What India watches; 2020. TV Universe Estimates 2020.

[12] Arora M Reddy KS Stigler MH Perry CL . Associations between tobacco marketing and use among urban youth in India. Am J Health Behav. 2008;32(3):283-294. 18067468 10.5555/ajhb.2008.32.3.283 PMC2830491

[13] Wakefield M Bayly M Durkin S , et al. Smokers’ responses to television advertisements about the serious harms of tobacco use: Pre-testing results from 10 low-to middle-income countries. Tobac Control. 2013;22(1):24-31. 10.1136/tobaccocontrol-2011-050171 21994276

[14] Arora M Mathur N Gupta VK Nazar GP Reddy KS Sargent JD . Tobacco use in Bollywood movies, tobacco promotional activities and their association with tobacco use among Indian adolescents. Tobac Control. 2012;21(5):482-487. 10.1136/tc.2011.043539 PMC3420563 21730099

[15] McKay AJ Negi NS Murukutla N , et al. Trends in tobacco, alcohol and branded fast-food imagery in Bollywood films, 1994-2013. PLoS One. 2020;15(5):e0230050. 32469942 10.1371/journal.pone.0230050 PMC7259671

[16] Murukutla N Turk T Prasad C , et al. Results of a national mass media campaign in India to warn against the dangers of smokeless tobacco consumption. Tobac Control. 2012;21(1):12-17. 10.1136/tc.2010.039438 21508418

[17] Murukutla N Bayly M Mullin S Cotter T Wakefield M Team IA-SARS . Male smoker and non-smoker responses to television advertisements on the harms of secondhand smoke in China, India and Russia. Health Educ Res. 2015;30(1):24-34. 25122618 10.1093/her/cyu044

[18] World Health Organization . Tobacco Free Initiative (TFI): World No Tobacco Day; 2013.

[19] Murukutla N Yan H Wang S , et al. Cost-effectiveness of a smokeless tobacco control mass media campaign in India. Tobac Control. 2018;27(5):547-551. 10.1136/tobaccocontrol-2016-053564 28798263

[20] Shah PB Pednekar MS Gupta PC Sinha DN . The relationship between tobacco advertisements and smoking status of youth in India. Asian Pac J Cancer Prev. 2008;9(4):637-642. 19256752

[21] Freeman B Watts C Astuti PAS . Global Tobacco Advertising, Promotion and Sponsorship Regulation: What’s Old, What’s New and where to Next? BMJ Publishing Group Ltd; 2022. 10.1136/tobaccocontrol-2021-056551 35241591

[22] World Health Organization . Smoke-free Movies: From Evidence to Action. Geneva: World Health Organization; 2009.

[23] Nazar GP Arora M Sharma N , et al. Changes in tobacco depictions after implementation of tobacco-free film and TV rules in Bollywood films in India: a trend analysis. Tob Control. 2021. 10.1136/tobaccocontrol-2021-056629 34312318

[24] Yadav A Glantz SA . Tobacco imagery in entertainment media: Evolution of tobacco-free movies and television programmes rules in India. BMJ Glob Health. 2021;6(1):e003639. 10.1136/bmjgh-2020-003639 PMC7786799 33402376

[25] Vital Strategies and World Health Organization . Evaluation of Tobacco Free Film and Television Policy in India; 2017.

[26] Kulkarni MM Kamath VG Cranwell J , et al. Assessment of tobacco imagery and compliance with tobacco-free rules in popular Indian films. Tobac Control. 2020;29(1):119-121. 10.1136/tobaccocontrol-2018-054613 PMC6952842 30772828

[27] Broadcast Audience Research Council (BARC) India . TV universe estimates 2020. 2020.

[28] TAM I . TAM Annual Universe Update; 2015.

[29] Mehta CR Patel NR . IBM SPSS Exact Tests. Armonk, NY: IBM Corporation; 2011.

[30] Yadav A Glantz SA . The Development and Implementation of Tobacco-free Movie Rules in India; 2020.

[31] Press Trust of India . ‘Ramadoss’s comments on film industry juvenile’. India Today. 2008.

[32] Minakshi Saini NS . Smoking on Screen Becomes a Trouble for Bollywood. Hindustan Timess. 2011.

[33] Refuse to Get Bullied on Anti-Smoking Warning in ‘Ugly’: Anurag Kashyap . Deccan Chronicale. 2013.

[34] In the High Court of Judicature at Bombay Ordinary Original Civil Juristiction. Tobacco Control Laws . WRIT PETITION NO. 119 OF 2014 Web site. https://www.tobaccocontrollaws.org/litigation/decisions/anurag-kashyap-v-union-of-india

[35] World Health Organization . Smoke-free Movies: From Evidence to Action; 2015.

[36] Durkin SJ Biener L Wakefield MA . Effects of different types of antismoking ads on reducing disparities in smoking cessation among socioeconomic subgroups. American journal of public health. 2009;99(12):2217-2223. 19833980 10.2105/AJPH.2009.161638 PMC2775761

[37] Durkin S Brennan E Wakefield M . Mass media campaigns to promote smoking cessation among adults: An integrative review. Tobac Control. 2012;21(2):127. 10.1136/tobaccocontrol-2011-050345 22345235

[38] Sly DF Arheart K Dietz N , et al. The outcome consequences of defunding the Minnesota youth tobacco-use prevention program. Prev Med. 2005;41(2):503-510. 15917046 10.1016/j.ypmed.2004.11.027

[39] Wakefield MA Spittal MJ Yong HH Durkin SJ Borland R . Effects of mass media campaign exposure intensity and durability on quit attempts in a population-based cohort study. Health Educ Res. 2011;26(6):988-997. 21730252 10.1093/her/cyr054 PMC3219882

[40] Wakefield MA Durkin S Spittal MJ , et al. Impact of tobacco control policies and mass media campaigns on monthly adult smoking prevalence. Am J Public Health. 2008;98(8):1443-1450. 18556601 10.2105/AJPH.2007.128991 PMC2446442

[41] IARC Handbooks of Cancer Prevention . Tobacco Control. France: Lyo. 2008.

[42] McGee R Ketchel J . Tobacco imagery on New Zealand television 2002-2004. Tob Control. 2006;15(5):412-414. 16998178 10.1136/tc.2006.016048 PMC2563655

[43] Marsh L McGee R Robertson L Ward M Llewellyn R . Little change in tobacco imagery on New Zealand television: 10 years on. Aust N Z J Publ Health. 2016;40(3):218-220. 10.1111/1753-6405.12527 27242253

[44] Barker AB Whittamore K Britton J Cranwell J . Content analysis of tobacco content in UK television. Tobac Control. 2019;28(4):381-385. 10.1136/tobaccocontrol-2018-054427 PMC6589448 30104409

[45] Barker AB Smith J Hunter A Britton J Murray RL . Quantifying tobacco and alcohol imagery in Netflix and Amazon Prime instant video original programming accessed from the UK: A content analysis. BMJ Open. 2019;9(2):e025807. 10.1136/bmjopen-2018-025807 PMC6398653 30765410

[46] New report from Truth Initiative® illustrates alarming rise of tobacco use in streaming content [press release] . Truth initiative. 2018.

[47] Arora M Nazar GP Chugh A , et al. Tobacco imagery in on-demand streaming content popular among adolescents and young adults in India: implications for global tobacco control. Tobac Control. 2021;30(1):42-48. 10.1136/tobaccocontrol-2019-055360 32273433

[48] Sharma P . Govt Issues New Anti-tobacco Rules for OTT Platforms; Mint. 2023.

[49] Basuroy T . Share in preferred film genres among Indians in 2018. 2021.

[50] Lyons A McNeill A Britton J . Tobacco imagery on prime time UK television. Tobac Control. 2014;23(3):257-263. 10.1136/tobaccocontrol-2012-050650 PMC3995275 23479113

[51] Truth Initiative. Tobacco’s Starring Role . How On-Screen Tobacco Imagery Drives Youth E-Cigarette Use and what the Entertainment Industry Can Do to Change the Picture; 2022.

[52] Broadcast Audience Research Council (BARC) India . WHAT YOUNG INDIA WATCHES!; 2018.

[53] Davis RM Gilpin EA Loken B Viswanath K Wakefield MA . The role of the media in promoting and reducing tobacco use. Health. 1998;98:4302.

[54] Thrasher JF Sargent JD Huang L Arillo-Santillán E Dorantes-Alonso A Pérez-Hernández R . Does film smoking promote youth smoking in middle-income countries? A Longitudinal study among Mexican adolescents. Cancer Epidemiol Biomarkers Prev. 2009;18(12):3444-3450. 19959694 10.1158/1055-9965.EPI-09-0883 PMC3837702

[55] Sargent JD Stoolmiller M Worth KA , et al. Exposure to smoking depictions in movies: Its association with established adolescent smoking. Arch Pediatr Adolesc Med. 2007;161(9):849-856. 17768284 10.1001/archpedi.161.9.849

