# Supplementary material for: An Observational Study of the Implementation of the Tobacco-Free Film and Television Policy in India
Source: Tob Use Insights. 2023 Oct 19;16:1179173X231205377. doi: 10.1177/1179173X231205377 (PMC10588424; doi:10.1177/1179173X231205377)
Supplement: Supplemental Material - An Observational Study of the Implementation of the Tobacco-Free Film and Television Policy in India [file sj-pdf-1-tui-10.1177_1179173X231205377.pdf]

Appendix 1: Amendments to COTPA, 2004 (Section 5)

MINISTRY OF HEALTH AND FAMILY WELFARE

(Department of Health and Family Welfare)

NOTIFICATION

New Delhi, the 21st September, 2012

GS.R 708(E).-ln exercise of the powers conferred by Section 31 of the Cigarettes and other Tobacco Products (Prohibition of Advertisement and Regulation of Trade and Commerce, Production, Supply and Distribution) Act, 2003 (34 of 2003), the Central Government hereby makes the following rules further to amend the Cigarettes and other Tobacco Products (Prohibition of Advertisement and Regulation of Trade and Commerce, Production, Supply and Distribution) Rules, 2004, namely:-

1. (1) These rules may be called the Cigarettes and other Tobacco Products (Prohibition of Advertisement and Regulation of Trade and Commerce, Production, Supply and Distribution) Amendment Rules, 2012.

   (2) They shall come into force from the 2nd Day of October, 2012.

2. In the Cigarettes and other Tobacco Products (Prohibition of Advertisement and Regulation of Trade and Commerce, Production, Supply and Distribution) Rules, 2004,-

   (a) for rule 7 the following rule shall be substituted, namely,-

"7, Health Spots and Message in Old Films and Television Programmes, displaying Tobacco Produce or their use.-

(1) The owner or manager of a cinema hall or theatre screening old films (Indian and foreign) which display tobacco products or their use, shall ensure that anti-tobacco health spots of minimum thirty seconds duration each are screened at the beginning and middle of the film:

Provided that such health spots shall be made available to the Central Board of Film Certification by the Ministry of Health and Family Welfare, Government of India.

(2) If the owner or manager of cinema hall or theatre fails to comply with the provision of sub-rule (1), the license of such cinema hall or theatre may be cancelled or suspended by the competent authority, after giving a reasonable opportunity to explain such failure.

(3) The broadcaster of old television programmes (including old Indian and foreign films) displaying tobacco products or their use, shall ensure that, -

(a) anti-tobacco health spots, of minimum thirty seconds duration each are screened at the beginning and the middle of the television programme:

Provided that such health spots shall be made available to the Central Board of Film Certification by the Ministry of Health and Family Welfare, Government of India;

(b) anti-tobacco health warning as a prominent static message is displayed at the bottom of the television screen during the period of display of the tobacco products or their use in the television programmes:

Provided that the anti-tobacco health warning message shall be legible and readable with font in black colour on white background, and, -

(i) with the warnings "Smoking causes cancer" or "Smoking kills" for smoking forms of tobacco use;

(ii) with the warnings "Tobacco causes cancer" or "Tobacco kills" for chewing and other smokeless forms of tobacco;

(iii) or with such other warnings as may be specified by the Central Government from time to time:

Provided that the anti-tobacco health warning message or health spots shall be in the same language as used in the film or television programme and in case of dubbed or sub-titled films or television programmes, the message or spots shall be carried in the language of dubbing or sub-title.

(4) If the broadcaster of old television programmes fails to comply with the provisions of sub-rule (3), the competent authority in the Ministry of Information and Broadcasting, Government of India shall take appropriate punitive action including cancellation or suspension of the license issued to such broadcaster, after giving a reasonable opportunity to explain such failure.

Explanation.-For tile purpose of this rule, -

(i) all films that receive Central Board of Film Certification prior to the effective date of this notification shall be categorized as "old films";

(ii) all television programmes produced prior to coming into effect of this notification shall be categorized as "Old Television Programme";

(iii) the expression "foreign film" implies "Imported" as defined in the Cinematograph (Certification) Rules, 1983;

(iv) the expression “television programmes” implies “programme” as defined in the Cable Television Networks (Regulation) Act, 1995”.

(b) For rule 8 the following rule shall be substituted, namely.-

"8. Health Spots, Message, and Disclaimers in New Films and Television Programmes.- (1) All new Indian or foreign films and television programmes displaying tobacco products or its use shall have,-

(a) a strong editorial justification explaining the necessity of the display of the tobacco products or their use in the film, to the Central Board of Film Certification;

(b) anti-tobacco health spots, of minimum thirty seconds duration each at the beginning and middle of the films and television programmes;

(c) anti-tobacco health warning as a prominent static message at the bottom of the screen during the period of display of the tobacco products or their use in the film and television programme;

(d) an audio-visual disclaimer on the ill-effects of tobacco use, of minimum twenty seconds duration each, in the beginning and middle of the film and television programme;

Provided that such health spots and disclaimer shall be made available to the Central Board of Film Certification by the Ministry of Health and Family Welfare, Government of India:

Provided further that no film displaying tobacco products or their use shall be certified for public exhibition by the Central Board of Film Certification unless the conditions specified in clauses (a) to (d) of sub-rule (1) are fulfilled.

(2) If the owner or manager of a cinema hall or theatre fails to comply with the provisions of sub-rule (1), the license of such cinema hall or theatre may be cancelled or suspended by the competent authority, after giving a reasonable opportunity to explain such failure;

(3) If the broadcaster of the television programmes fails to comply with the provisions of clauses (b) to (d) of sub-rule (1), the competent authority in the Ministry of Information and Broadcasting, Government of India shall take appropriate punitive action including cancellation or suspension of the license issued to such broadcaster, after giving a reasonable opportunity to explain such failure.

(4) The anti-tobacco health warning message as specified in clause (c) of sub-rule (1) shall be legible and readable, with font in black colour on white background, and, -

(i) with the warnings “Smoking causes cancer” or “Smoking kills” for smoking forms of tobacco use;

(ii) with the warnings “Tobacco causes cancer” or “Tobacco kills” for chewing and other smokeless forms of tobacco;

(iii) or with such other warnings as may be specified by the Central Government from time to time.

(5) The anti-tobacco health warning messages or health spots shall be in the same language as used in the film or television programme and in case dubbed or sub-titled films or television programmes, the messages or spots shall be carried in the language of dubbing or sub-title/

Explanation. – For the purpose of this rule, -

(i) all films that receive Central Board of Film certification after the effective date of this notification shall be categorized as “New Films”.

(ii) all television programmes produced after the effective date of this notification shall be categorized as “New Television Programme”.

[F.No. P016012/1/2005-PH]

[F.No. P-16-12/1/2005-PH]

Smt. SHAKUNTALA D. GAMLIN, Jt. Secy.

Note: The principal rules were published in the Gazette of India, vide notification number G.S.R. 1377 dated the 25th February, 2004, and were subsequently amended vide notification number G.S.R. 345(E) dated the 31st May, 2005, vide G.S.R. 698(E) dated 30th November, 2005, vide G.S.R. 656(E), dated 20th October, 2006 and vide G.S.R 785 dated 27th October, 2011.

Appendix 2

Table 1. Sampling: Selection of Channels by Type and Gross Rating Points

	National	International	Regional
**High GRP**	Zee tv	Discovery channel	Z telugu
	Movies ok	History tv18	Colors kannada
	Colors viacom18	Hbo	Jalsha movies
	Utv action	Id investigation discovery	Isaiaruvi
	Sony max	Tlc	Tarang
**Medium GRP**	9x jalwa	Nick jr.nickelodeon	Maiboli
	Filmy	Star world	Kiran tv
	Sahara one	Star movies action	Mahuaa
	Zindagi	Vh1	Rupashi bangla
	Star plus hd	Star movies hd	Amrita
**Low GRP**	Z hd cinema	Fox life hd	Sun tv hd
	Life ok hd	Mn+ hd	We
	Sony entertainment tv hd	Travel xp hd	Kappa tv
	&Tvhd	Colors infinity hd	Gemini hd
	Z hd studio	Axn hd	Makkal tholaikatchi

Table 2. Number of Movie Theaters and Exit Surveys Conducted in Selected Cities in India

		**Zones**						
		**North. (n=73)**	**East (n=81)**	**West (n=79)**	**South (n=75)**	**Total Movie Theaters (n=308)**	**Surveys (n=10 per theater)**	**Total Surveys (n=3,080)**
[illegible]	Metro	Delhi	Kolkata	Mumbai	Bangalore	167	10	**1670**

	Tier 1	Ludhiana	Patna	Indore	Warangal	49	10	**490**
		Jaipur	Ranchi	Pune	Vishakhapatnam	51	10	**510**
	Smaller	Chandigarh	Muzaffarpur	Gwalior	Guntur	21	10	**210**
		Gurgaon	Siliguri	Kolhapur	Mysore	20	10	**200**

Appendix 3

Evaluation of the Implementation of Film Rule in Movie Theatres

Observation Questionnaire

**Interviewer Details**		
**Interviewer Name:**	**Interviewer Code:**	
**Supervisor Name:**	**Supervisor Code:**	
**Questionnaire Serial Number:**		
**Scrutiny By:**	**Date:**	
**Data Entry By:**	**Date:**	
**Interviewer verification:**		

INTERVIEWER SECTION

This section is to be completed by the interviewer alone

**Movie hall name:**				
**Movie hall address:**				
**Date:**				
**Movie name:**				
**Show Time:**		Start Time:		
		End Time:		
**Observations**		**Options**	**Codes**	**Instructions**
A1.	In which Audi/Screen was the	Not Applicable	1	

	movie seen?	Screen 1	2	
		Screen 2	3	
		Screen 3	4	
		Screen 4	5	
		Screen 5	6	
		Screen 6	7	
		Screen 7	8	
		Screen 8	9	
A2.	What was the genre of the movie?	Action	1	
		Adventure	2	
		Comedy	3	
		Romantic	4	
		Thriller	5	
		Any other	__________(99)	Please specify
A3.	What is the Movie Rating provided by Film board?	U	1	
		U/A	2	
		A	3	
A4.	In what language was the movie shown?	Hindi	1	
		English	2	
		Bangla	3	
		Assamese	4	
		Panjabi	5	
		Gujarati	6	
		Dogri	7	
		Kannada	8	
		Marathi	9	

		Tamil	10	
		Telugu	11	
		Malayalam	12	
		Oriya	13	
		Any other Language	_________(99)	Please specify

Observations during the movie

		Options	Codes	Instructions
A5.	Was the anti-tobacco disclaimer played?<br><br>*[Instruction: Please note that when films are dubbed or subtitled, the disclaimer texts should be in the same language as the language of the dubbing or subtitle]*	Yes, in the same language as the language of the movie	1	
		Yes, but not in the same language as the language of the movie	2	
		No	3	
A6.	Was the anti-tobacco PSA 1 shown before the film?<br><br>*[Instruction: Please note that when films are dubbed or subtitled, the PSA should be in the same language as the language of the dubbing or subtitle]*	Yes, in the same language as the language of the movie	1	
		Yes, but not in the same language as the language of the movie	2	
		No	3	
A7.	Was the anti-tobacco PSA 2 shown before the film?<br><br>*[Instruction: Please note that when films are dubbed or subtitled, the PSA*	Yes, in the same language as the language of the movie	1	
		Yes, but not in the	2	

	should be in the same language as the language of the dubbing or subtitle]	same language as the language of the movie		
		No	3	
A8.	Was the anti-tobacco PSA 1 shown during the intermission?<br><br>*[Instruction: Please note that when films are dubbed or subtitled, the PSA should be in the same language as the language of the dubbing or subtitle]*	Yes, in the same language as the language of the movie	1	
		Yes, but not in the same language as the language of the movie	2	
		No	3	
A9.	Was the anti-tobacco PSA 2 shown during the intermission?<br><br>*[Instruction: please note that when films are dubbed or subtitled, the PSA should be in the same language as the language of the dubbing or subtitle]*	Yes, in the same language as the language of the movie	1	
		Yes, but not in the same language as the language of the movie	2	
		No	3	
A10.	Total number of tobacco consumption (smoking or smokeless tobacco) scenes in the movie	☐☐		
A11.	Total number of times the running scroll was shown in the movie	☐☐		**GO TO A18 IF NO RUNNING SCROLL SHOWN**
**ASK A12 – A17 ONLY IF RUNNING SCROLL SHOWN ATLEAST ONCE IN THE MOVIE**				
A12.	Was the running scroll shown in the same language as the language of the movie?	Yes	1	

	[Instruction: Please note that when films are dubbed or subtitled, the running scroll texts should be in the same language as the language of the dubbing or subtitle]	No	2	
A13.	Where was the running scroll placed in the movie?	Top	1	
		Middle	2	
		Bottom	3	
A14.	What was the position of running scroll?	Left	1	
		Centre	2	
		Right	3	
A15.	Was the font of running scroll black in color?	Yes, always	1	
		Yes, sometimes	2	
		Never	3	
A16.	Was the running scroll shown on white background?	Yes, always	1	
		Yes, sometimes	2	
		Never	3	
A17.	What was the message shown in the running scroll?	Smoking causes cancer	1	
		Smoking kills	2	
	[Instruction: Multiple coding possible in case more than 1 running scrolls were shown in movie]	Tobacco causes cancer	3	
		Tobacco kills	4	
		Others:__________ ____________	99	Please specify
A18.	Any other health disclaimers shown before or during the movie and what was it about? (please specify)	----------------------	99	Please specify

Appendix 4

Exit Survey Questionnaire

Questionnaire Number			
**Introduction and Consent**			

Greetings (Namaste)

My name is _______________________ and I work for Kadence International, a research organization. We conduct studies on various social issues and on consumer products. We are conducting this study on behalf of World Lung Foundation, which is an NGO. In this study we are asking people for their opinion on issues that affect our society.

About this consent form:

This form provides important information about participation in a research study and seeks your consent for participation. As a prospective research participant, you have the right to take your time in deciding about participating in this research and you are encouraged to discuss your decision to participate with your family. If you have any questions about the research or any portion of this form, please ask us.

Purpose of this research

We are conducting this Viewership Study to understand people's experiences while viewing films. We expect to talk to 3000 movie viewers across India. We will be asking you questions about what you just watched and your reactions to what you watched.

Participation is voluntary

Participation in this study is entirely voluntary: You may change your mind and leave the study at any time without any prejudice or penalty. It is entirely your choice whether or not to take part in this research.

Benefits and risks

The results of this study will be used to understand people's viewing experiences and will make an important social contribution.

Since this is a public opinion survey and does not require disclosure of any personal information or participation in any activities, we do not expect any risk to you from your participation. You will not be paid to participate in this study.

Data confidentiality

If you decide to participate in this research study, please be assured that your data will be kept completely confidential. Only the researchers and the sponsors of this study will have access to the data that identifies you. Identifiable information will not be published or shared with any other third party. All data will be reported in the aggregate and you will never be identified in any reports, presentations, or communications based on this study.

May I begin the interview now?

**YES**, RESPONDENT AGREES TO BE INTERVIEWED.	**BEGIN INTERVIEW**
**NO**, RESPONDENT DOES NOT AGREE TO BE INTERVIEWED.	**END INTERVIEW**

Statement of Consent:

I have read/ or been read the information in this consent form including risks and possible benefits. All my questions about the research study have been answered to my satisfaction.

I consent to participate in the study. I authorize the use and disclosure of my information to the parties listed in the privacy section of this consent form.

Signature of Interviewer: ___________________________

Date: _________________________________

RESPONDENT SECTION

**SECTION A: DEMOGRAPHICS (*Ask all qualified respondents)***				
**Q. No.**	**Questions**	**Options**	**Codes**	**Instructions**
A1.	GENDER<br><br>*[Do not ask observe and record]*	Male	1	
		Female	2	
A2.	Did you just watch a movie in this theater?	Yes	1	
		No	2	→ **Terminate**
A3.	What was the name of the movie?<br><br>*[Record Verbatim]*	____________________________		
A4.	In which Audi/Screen did you see the	Not Applicable	1	

	movie?<br><br>*[Interviewer to Check Ticket*<br><br>*Single Coding Only]*	Audi/Screen 1	2	→ **Terminate if not matching sampled Audi/ Screen**
		Audi/Screen 2	3	
		Audi/Screen 3	4	
		Audi/Screen 4	5	
		Audi/Screen 5	6	
		Audi/Screen 6	7	
		Audi/Screen 7	8	
		Audi/Screen 8	9	
A5.	What is the month and year of your birth?<br><br>*[If DON'T KNOW for month "99" For year 9999]*	_ _ / _ _ _ _<br><br>**Month Year**		
A6.	Do you <u>currently</u> smoke tobacco?<br><br>*[Single Coding Only]*	Yes, daily	1	
		Yes, less than daily/occasionally/socially	2	
		No, I do not smoke	3	→**A8**
A7.	How many cigarettes/bidis/others do you smoke in a day?<br><br>*[Single Coding Only]*	10 or less	1	
		11 – 20	2	
		21 – 30	3	
		31 or more	4	
		Don't know/Can't say	99	
A8.	In the <u>past</u>, have you smoked tobacco on a daily basis, less than daily, or not at all?<br><br>*[Single Coding Only]*	Daily	1	
		Less than Daily	2	
		Not at All	3	

A9.	Do you <u>currently</u> use smokeless tobacco (betel quid with tobacco, *sada/surti*, *khaini*	Yes, daily	1	→**SEC B**
		Yes, less than	2	

	or tobacco lime mixture, *gutkha*?)	daily/occasionally/socially		
	[Single Coding Only]	No, I do not chew tobacco	3	
A10.	In the past, have you used smokeless tobacco on a daily basis, less than daily, or not at all?	Daily	1	
		Less than Daily/occasionally/socially	2	
	[Single Coding Only]	Not at All	3	

**SECTION B: TOBACCO CONTROL MESSAGES (*Ask all qualified respondents)***					
**Q. No.**	**Questions**	**Options**		**Codes**	**Instructions**
B1.	During the movie you just watched, did you come across any advertisements or messages about…				
		Yes	No	DK	
i	Anti-Tobacco (smoking/ smokeless tobacco) and health	1	2	9	
ii	Anti-Alcohol	2	2	9	
iii	Cleanliness	1	2	9	
iv	Vaccination	1	2	9	
v	Other (specify): ____				
B2.	In this movie, did you notice ANY of the characters in the film doing the following?				
		Yes	No	DK	
i	Using tobacco, including smoking or chewing tobacco	1	2	9	
ii	Consuming alcohol consumption	1	2	9	

B3.	***Ask if Coded "1" in B2B2.i***	Positive	1	

	You said that you noticed tobacco being consumed by characters in the movie. Would you say that the use of tobacco was shown in a positive way, negative way or in a neutral way?<br><br>*[Single Coding Only]*	Negative	2	
		Neutral/ mixed	3	

**SECTION C: KNOWLEDGE, ATTITUDE, AND BEHAVIORS ON TOBACCO (*Ask question C1 to C4 to only current tobacco users coded "1" or "2" in A6 or A9 and ask C5 onwards to all respondents)***				
**Q. No.**	**Questions**	**Options**	**Codes**	**Instructions**
C1.	When did you last use tobacco (smoke or chew) before entering the theater?<br><br>*[Single Coding Only]*	5 minutes	1	
		6-30 minutes	2	
		31-60 minutes	3	
		More than 60 minutes	4	
C2.	How likely or unlikely are you to quit using tobacco (smoking or chewing) permanently?<br><br>*Encourage the best guess.*<br><br>*[Single Coding Only]*	Definitely will	1	
		Very likely	2	
		Quite likely	3	
		50/50	4	
		Quite unlikely	5	
		Very unlikely	6	
		Definitely will not	7	
C3.	Do you feel good or bad about being using tobacco, or do you have mixed feelings?<br><br>*[Single Coding Only]*			

	Very good (positive) feelings about being a smoker		1	
	Somewhat Good (positive) feelings about being a smoker		2	
	Neither good nor bad feelings about being a smoker		3	
	Somewhat bad (negative) feelings about being a smoker		4	
	Very bad (negative) feelings about being a smoker		5	
C4.	What do you think is the likelihood of becoming ill from tobacco if you continue to use it?<br><br>**[***Single Coding Only]*	Not at all likely	1	
		Somewhat likely	2	
		Probably Likely	3	
		Very likely	4	
		Definite/ Certain to happen	5	

Now I am going to read to you some phrases about tobacco and tobacco smoke. Please tell me how much you agree or disagree with each one of them.

Read statements to all respondents.

		Strongly Disagree	Disagree	Neither Agree Nor Disagree	Agree	Strongly Agree
C5.	The people important to me believe that I shouldn't use tobacco	1	2	3	4	5
C6.	Smoking causes serious illnesses to the smoker.	1	2	3	4	5
C7.	I disapprove of smoking	1	2	3	4	5
C8.	Exposure to smoke from another person's cigarette (or passive smoking) causes serious illnesses in non-smokers.	1	2	3	4	5
C9.	I am concerned for my health when someone is smoking near me	1	2	3	4	5
C10.	The law that prohibits smoking in indoor places will benefit the public's health.	1	2	3	4	5

SECTION D: IMPACT OF TOBACCO CONTROL MEASURES (*Ask All)*

Q. No.	Questions	Options	Codes	Instructions
D1.	Did you see this anti-tobacco advertisement either before or during the movie you just watched?<br><br>*[Interviewer to present showcard with the image from ad 1]*	Yes	1	
		No	2	→D21
		Don't Remember	8	

How strongly do you disagree or agree with the following statements about this AD?<br><br>**READ OUT ALL THE STATEMENTS ONE-BY-ONE**		Strongly Disagree	Disagree	Neither Agree Nor Disagree	Agree	Strongly Agree
D2.	It was EASY to understand	1	2	3	4	5
D3.	It made me STOP and THINK.	1	2	3	4	5
D4.	It was RELEVANT to me and my life	1	2	3	4	5
D5.	It provided new information to me.	1	2	3	4	5
D6.	It made me feel CONCERNED about the health effects of tobacco	1	2	3	4	5
D7.	I am likely to talk about this advertisement to other people.	1	2	3	4	5
D8.	*[IF A NON-USER (coded "3" in A6 and "3" in A9)]*<br><br>It made me more likely to AVOID using tobacco	1	2	3	4	5
D9.	*[IF A tobacco user (coded "1" or "2" in A6 or A9)]*<br><br>It made me more likely to quit tobacco	1	2	3	4	5
D10.	*[IF A SMOKER (coded "1" or "2" in A6)]*<br><br>It made me more likely to avoid exposing others to smoke from my cigarette/bidi	1	2	3	4	5

D11.	Did you see this anti-tobacco advertisement either before or during the movie you just watched?<br><br>*[Interviewer to present showcard with the image from ad 2]*	Yes	1	
		No	2	→D21
		Don't Remember	8	→D21

How strongly do you disagree or agree with the following statements about this AD?<br><br>**READ OUT ALL THE STATEMENTS ONE-BY-ONE**		Strongly Disagree	Disagree	Neither Agree Nor Disagree	Agree	Strongly Agree
D12.	It was EASY to understand	1	2	3	4	5
D13.	It made me STOP and THINK.	1	2	3	4	5
D14.	It was RELEVANT to me and my life	1	2	3	4	5
D15.	It provided new information to me.	1	2	3	4	5
D16.	It made me feel CONCERNED about the health effects of tobacco	1	2	3	4	5
D17.	I am likely to talk about this advertisement to other people.	1	2	3	4	5
D18.	*[IF A NON-USER (coded "3" in A6 and "3" in A9)]*<br><br>It made me more likely to AVOID using tobacco	1	2	3	4	5
D19.	*[IF A tobacco user (coded "1" or "2" in A6 or A9)]*<br><br>It made me more likely to quit tobacco	1	2	3	4	5
D20.	*[IF A SMOKER (coded "1" or "2" in A6)]*<br><br>It made me more likely to avoid exposing others to smoke from my cigarette/bidi	1	2	3	4	5

D21.	Did you see this disclaimer either before or during the movie you just watched?<br><br>*[Interviewer to present showcard with the image from disclaimer]*	Yes	1	
		No	2	→D31
		Don't Remember	8	→D31

How strongly do you disagree or agree with the following statements about the DISCLAIMER?<br><br>**READ OUT ALL THE STATEMENTS ONE-BY-ONE**		Strongly Disagree	Disagree	Neither Agree Nor Disagree	Agree	Strongly Agree
D22.	It was EASY to understand	1	2	3	4	5
D23.	It made me STOP and THINK.	1	2	3	4	5
D24.	It was RELEVANT to me and my life	1	2	3	4	5
D25.	It provided new information to me.	1	2	3	4	5
D26.	It made me feel CONCERNED about the health effects of tobacco	1	2	3	4	5
D27.	I am likely to talk about this disclaimer to other people.	1	2	3	4	5
D28.	*[IF A NON-USER (coded "3" in A6 and "3" in A9)]*<br><br>It made me more likely to AVOID using tobacco	1	2	3	4	5
D29.	*[IF A tobacco user (coded "1" or "2" in A6 or A9)]*<br><br>It made me more likely to quit tobacco	1	2	3	4	5
D30.	*[IF A SMOKER (coded "1" or "2" in A6)]*<br><br>It made me more likely to avoid exposing others to smoke from my cigarette/bidi	1	2	3	4	5

D31.	Do you recall seeing this running scroll appear on the screen during the film?<br><br>*[Interviewer to present showcard with the image from running scroll]*	Yes	1	
		No	2	→SEC E
		Don't Remember	8	→SEC E

How strongly do you disagree or agree with the following statements about this RUNNING SCROLL?<br><br>***SHOW IMAGE CARD AGAIN. READ OUT ALL THE STATEMENTS ONE-BY-ONE***		Strongly Disagree	Disagree	Neither Agree Nor Disagree	Agree	Strongly Agree
D32.	It was EASY to understand	1	2	3	4	5
D33.	It made me STOP and THINK.	1	2	3	4	5
D34.	It was RELEVANT to me and my life	1	2	3	4	5
D35.	It provided new information to me.	1	2	3	4	5
D36.	It made me feel CONCERNED about the health effects of tobacco	1	2	3	4	5
D37.	I am likely to talk about this running scroll to other people.	1	2	3	4	5
D38.	*[IF A NON-USER (coded "3" in A6 and "3" in A9)]*<br><br>It made me more likely to AVOID using tobacco	1	2	3	4	5
D39.	*[IF A tobacco user (coded "1" or "2" in A6 or A9)]*<br><br>It made me more likely to quit tobacco	1	2	3	4	5
D40.	*[IF A SMOKER (coded "1" or "2" in A6)]*<br><br>It made me more likely to avoid exposing others to smoke from my cigarette/bidi	1	2	3	4	5

**SECTION E: EDUCATION AND SEC (*<u>Ask All</u>*)**				
**Q. No.**	**Questions**	**Options**	**Codes**	**Instructions**
E1.	What is the highest level of education you	No formal schooling	1	

	have completed?<br><br>*[Single Coding ONLY]*	Less than primary school completed	2	
		Primary school completed	3	
		Less than secondary school completed	4	
		Secondary school completed	5	
		High school completed	6	
		College/university completed	7	
		PG degree completed	8	
		Don't know/Can't Say	99	

E2.	Do you have the following in your HH?	Yes	No	
i	Electricity	1	2	
ii	Flush toilet	1	2	
iii	Fixed telephone	1	2	
iv	Cell telephone	1	2	
v	Television	1	2	
vi	Radio/Transistor/FM on Cell phone	1	2	
vii	Refrigerator	1	2	
viii	Car	1	2	
ix	Moped/scooter/motorcycle	1	2	
x	Washing Machine	1	2	

E3.	How many children under the age of 16 reside in this household?<br><br>[Record Verbatim]					

Appendix 5

Assessment of Implementation and Enforcement of Film Rule on Television

Observation Questionnaire

Interviewer Details			
**Interviewer Name:**	**Interviewer Code:**		
**Supervisor Name:**	**Supervisor Code:**		
**Questionnaire Serial Number:**			
**Scrutiny By:**	**Date:**		
**Data Entry By:**	**Date:**		
**Interviewer verification:**			

INTERVIEWER SECTION

This section is to be completed by the interviewer alone.

Section A: Channel and Show Information				
**S. No.**	**Information**	**Responses**	**Codes**	**Instructions**
A1	Date of observation	D D M M Y Y		
A2	Name of the channel	____________________		
A3	Channel unique number			
A4	Channel category<br><br>**Single Coding Only**	National	1	
		International	2	
		Regional	3	
A5	Channel sub-category<br><br>**Single Coding Only**	Entertainment	1	
		Movies	2	
		Music	3	
		Kids	4	
		Others	5	

A6	Show/movie name	____________________		
A7	Show/movie START TIME<br><br>**(Record in Hours and Minutes)**	H H : M M	AM/PM	
A8	Show/movie END TIME<br><br>**(Record in Hours and Minutes)**	H H : M M	AM/PM	
A9	Show/movie duration<br><br>**(Record in Hours and Minutes)**	H H : M M		
A10	**Note: Record this only in case of movies**<br><br>Central Board Film Certification (CBFC) rating<br><br>**Single Coding Only**	U (Universal)	1	
		U/A (Universal with adult themes)	2	
		A (Adult only	3	
		S (Special Group)	4	
		Others (please specify………………)	99	

S. No.	Information	Responses	Codes	Instructions
A11	Show/ movie Language<br><br>**Single Coding Only**	Hindi	1	
		English	2	
		Bangla	3	
		Assamese	4	
		Punjabi	5	
		Gujarati	6	
		Kannada	7	
		Marathi	8	
		Tamil	9	
		Telugu	10	
		Malayalam	11	

		Oriya	12	
		Bhojpuri	13	
		Others (please specify………………)	99	

Section B: Observation of Film Rule					
**S. No.**	**Questions**	**Responses**	**Codes**		**Instructions**
			**A. Before the show/ movie**	**B. During the show/ movie**	
**Anti-Tobacco Disclaimer**					
B1	Was anti-tobacco disclaimer shown before and during the show/ movie?<br><br>**Single Coding Only**	Yes, in the same language as the language of the show/ movie	1	1	→ **B2**
		Yes, but not in the same language as the language of the show/ movie	2	2	
		No	3	3	→ **B9**
B2	**Record duration only if 1 or 2 is coded in B1**<br><br>What was the duration of the disclaimer shown?<br><br>**Note: Record the duration in seconds using stopwatch**	**Record duration in seconds:**	☐☐	☐☐	
B3	**Code only if 1 or 2 is coded in B1**<br><br>What type of disclaimer was	Only Audio	1	1	→ **B9**
		Only Visual	2	2	→ **B4**
		Audio-visual	3	3	

	played?<br><br>**Single Coding Only**				

S. No.	Questions	Responses	Codes		Instructions
			A. Before the show/ movie	B. During the show/ movie	
B4	**Code only if 2 or 3 is coded in B3**<br><br>What kind of visual/ audio-visual disclaimer was shown?<br><br>**Single Coding Only**	Static visual (one frame)	1	1	
		Moving visual (multiple frames)	2	2	
B5	**Code only if 2 or 3 is coded in B3**<br><br>What was the background color on which disclaimer was shown?<br><br>**Single Coding Only**	Black	1	1	
		White	2	2	
		Others (please specify……………………)	99	99	
B6	**Code only if 2 or 3 is coded in B3**<br><br>What kind of warning was shown in the disclaimer?<br><br>**Single Coding Only**	Only graphical warning	1	1	**→ B9**
		Only textual warning	2	2	**→ B7**
		Both graphical and textual warning	3	3	

B7	**Code only if 2 or 3 coded in B6**<br><br>What was the color of the font that was used for textual warning in the disclaimer?<br><br>**Single Coding Only**	Black	1	1	
		White	2	2	
		Others (please specify……………………)	99	99	
B8	**Code only if 2 or 3 is coded in B6**<br><br>Was the contents of the textual message shown as per COTPA law?<br><br>**Note: Please see the show cards before coding.**<br><br>**Single Coding Only**	Yes	1	1	
		No	2	2	

S. No.	Questions	Responses	Codes		Instructions
			A. Before the show/ movie	B. During the show/ movie	
**Statutory Anti-Tobacco Health Warning**					
B9	Was any statutory anti-tobacco health warning shown before and during the show/ movie?<br><br>**Single Coding Only**	Yes, in the same language as the language of the show/ movie	1	1	**→ B10**
		Yes, but not in the same language as the language of the show/ movie	2	2	
		No	3	3	**→ B16**

B10	**Record duration only if 1 or 2 is coded in B9**<br><br>What was the duration of the statutory anti-tobacco health warning shown?<br><br>**Note: Record the duration in seconds using stopwatch**	**Record duration in seconds:**	☐☐	☐☐	
B11	**Code only if 1 or 2 is coded in B9**<br><br>What type of statutory anti-tobacco health warning was played?<br><br>**Single Coding Only**	Only Audio	1	1	**→ B16**
		Only Visual	2	2	**→ B12**
		Audio-visual	3	3	
B12	**Code only if 2 or 3 is coded in B11**<br><br>What kind of visual/ audio-visual statutory anti-tobacco health warning was shown?<br><br>**Single Coding Only**	Static visual (one frame)	1	1	
		Moving visual (multiple frames)	2	2	
B13	**Code only if 2 or 3 is coded in B11**<br><br>What was the background color on which statutory anti-tobacco health warning was shown?	Black	1	1	
		White	2	2	
		Red	3	3	
		Blue	4	4	
		Green	5	5	
		Yellow	6	6	

	**Single Coding Only**	Purple	7	7	
		Running Background (Multiple colors)	8	8	
		Others (please specify……………………)	99	99	

S. No.	Questions	Responses	Codes		Instructions
			A. Before the show/ movie	B. During the show/ movie	
B14	**Code only if 2 or 3 is coded in B11**<br><br>What kind of warning was shown in the statutory anti-tobacco health warning?<br><br>**Single Coding Only**	Only graphical warning	1	1	**→ B16**
		Only textual warning	2	2	**→ B15**
		Both graphical and textual warning	3	3	
B15	**Code only if 2 or 3 coded in B14**<br><br>What was the color of the font that was used for textual warning in the statutory anti-tobacco health warning?<br><br>**Single Coding Only**	Black	1	1	
		White	2	2	
		Red	3	3	
		Blue	4	4	
		Green	5	5	
		Yellow	6	6	
		Purple	7	7	
		Others (please specify……………………)	99	99	
**Public Service Announcement (PSA) 1**					
B16	Was PSA 1 shown before and during the show/ movie?	Yes, in the same language as the language of the show/	1	1	**→ B17**

	**Single Coding Only**	movie			
		Yes, but not in the same language as the language of the show/ movie	2	2	
		No	3	3	**→ B19**
B17	**Code only if 1 or 2 is coded in B16**<br><br>Which PSA was shown as PSA 1?<br><br>**Single Coding Only**	Dhuan	1	1	
		Child	2	2	
		Sponge	3	3	
		Mukesh	4	4	
		Surgeon	5	5	
		Cigarettes are eating you alive	6	6	
		Heartbreak	7	7	
		Artery (Bidi)	8	8	
		Artery (Cigarette)	9	9	
		Tears you apart	10	10	
		Sunita	11	11	
		Tobacco is eating your baby alive	12	12	
		Others (please specify)	99	99	

**S. No.**	**Questions**	**Responses**	**Codes**		**Instructions**
			**A. Before the show/ movie**	**B. During the show/ movie**	

B18	**Code only if 1 or 2 is coded in B16**<br><br>What was the duration of the PSA 1 shown?<br><br>**Note: Record the duration in seconds using stopwatch**	**Record duration in seconds:**	☐☐	☐☐	
**Public Service Announcement (PSA) 2**					
B19	Was PSA 2 shown before and during the show/ movie?<br><br>**Single Coding Only**	Yes, in the same language as the language of the show/ movie	1	1	**→ B20**
		Yes, but not in the same language as the language of the show/ movie	2	2	
		No	3	3	**→ B22**
B20	**Code only if 1 or 2 is coded in B19**<br><br>Which PSA was shown as PSA 2?<br><br>**Single Coding Only**	Dhuan	1	1	
		Child	2	2	
		Sponge	3	3	
		Mukesh	4	4	
		Surgeon	5	5	
		Cigarettes are eating you alive	6	6	
		Heartbreak	7	7	
		Artery (Bidi)	8	8	
		Artery (Cigarette)	9	9	
		Tears you apart	10	10	
		Sunita	11	11	
		Tobacco is eating your	12	12	

		baby alive			
		Others (please specify)	99	99	
B21	**Record only if 1 or 2 is coded in B19**<br><br>What was the duration of the PSA 2 shown?<br><br>**Note: Record the duration in seconds using stop watch**	**Record duration in seconds:**	☐☐	☐☐	

S. No.	Questions	Responses	Codes		Instructions
**Information on Tobacco Scenes**					
B22	Was any tobacco product displayed/ consumed in the show/ movie?<br><br>**Single Coding Only**	Yes	1		**→ B23**
		Not Clear	2		
		No	3		**→ C1**
			**A.**	**B.**	
B23	**Code only if 1 is coded in B22**<br><br>A. What kind of tobacco product/s was/ were displayed/ consumed in the show/ movie?<br><br>B. How many tobacco scenes	Cigarette	1	☐☐	**→ B25**
		Bidi	2	☐☐	
		Cigar	3	☐☐	
		Hukka	4	☐☐	
		Gutkha/ Khaini	5	☐☐	**→ B24**
		Pan	6	☐☐	

	were observed in the show/movie?<br><br>**Multiple Coding Possible**	Pan Masala	7	
		Pan Shop	8	
B24	**Code only if 5, 6, 7 or 8 is coded in B23**<br><br>In case of chewing tobacco, was the display clearly identifiable in the show/ movie?<br><br>**Single Coding Only**	Yes, always	1	
		Yes, sometimes	2	
		Never	3	

S. No.	Questions	Responses	Codes	Instructions
**Static Message**				
B25	**Code only if 1 or 2 is coded in B22**<br><br>Was the static message shown in the show/ movie when tobacco product was displayed/ consumed?<br><br>**Single Coding Only**	Yes, always	1	**→ B27**
		Yes, sometimes	2	**→ B26**
		No	3	**→ C1**
B26	**Code only if 2 is coded in B25**<br><br>If the static message was not shown every time on a tobacco scene, then how many times was the static message shown?	**Record total number:**		

B27	**Code only if 1 or 2 is coded in B25**<br><br>Whenever the static message was shown, was it in the same language as the language of the show/ movie?<br><br>**Single Coding Only**	Yes, always	1	
		Yes, sometimes	2	
		Never	3	
B28	**Code only if 1 or 2 is coded in B25**<br><br>What was/were the message/s shown in the static message /s shown in the show/ movie?<br><br>**Multiple Coding Possible**	Smoking causes cancer	1	
		Smoking kills	2	
		Tobacco causes cancer	3	
		Tobacco kills	4	
		Others (please specify…)	99	
B29	**Code only if 1 or 2 is coded in B25**<br><br>Was the static message shown on screen till tobacco product was displayed/ consumed in the show/ movie?<br><br>**Single Coding Only**	Yes, always	1	
		Yes, sometimes	2	
		Never	3	
B30	**Code only if 1 or 2 is coded in B25**<br><br>Was the static message readable?	Yes, every time	1	
		Yes, but not every time	2	
		No, it was never readable	3	

	**Single Coding Only**			
**S. No.**	**Questions**	**Responses**	**Codes**	**Instructions**
B31	**Code only if 1 or 2 is coded in B25**<br><br>What was the background on which static message/s was/ were shown?<br><br>**Multiple Coding Possible**	Black	1	
		White	2	
		No background, but the background of the show	3	
		Any other background color (please specify…)	99	
B32	**Code only if 1 or 2 is coded in B25**<br><br>What was the color of the font in which warning message/s was/ were written in static message/s?<br><br>**Multiple Coding Possible**	Black	1	
		White	2	
		Any other color (please specify…)	99	
B33	**Code only if 1 or 2 is coded in B25**<br><br>What was/ were the position/s of the static message/s shown on the screen?<br><br>**Multiple Coding Possible**	Top	1	
		Middle	2	
		Bottom	3	
B34	**Code only if 1 or 2 is coded in B25**	Right	1	
		Centre	2	

	What was/ were the placement/s of the static message/s shown on the screen?<br><br>**Multiple Coding Possible**	Left	3	

**Section C: Surrogate Advertisement**				
**S. No.**	**Questions**	**Responses**	**Codes**	**Instructions**
C1	Was any tobacco product/s or pan shop was shown in any scene of the show/ movie/advertisement?<br><br>**Multiple Coding Possible**<br><br>**Note: Check responses with question B23**	Cigarette	1	**→ C2**
		Bidi	2	
		Cigar	3	
		Hukka	4	
		Gutkha/ Khaini	5	
		Pan	6	
		Pan Masala	7	
		Pan Shop	8	
		Pan Masala or any other tobacco product advertisement	9	
		E-Cigarette	10	
		No, Nothing was shown	11	**→ D1**
C2	**Code only if anything between 1 to 10 is coded in C1**<br><br>Was the brand of tobacco product/s clearly visible?<br><br>**Single Coding Only**	Yes, every time	1	**→ C3**
		Yes, sometimes	2	
		Never	3	**→ D1**

C3	**Ask only if 1 or 2 is coded in C2**<br><br>Which brand/s was/were visible in the show/ movie? (please specify…)<br><br>**Multiple Response Possible**	1. ___________________<br>2. ___________________<br>3. ___________________<br>4. ___________________<br>5. ___________________		

**Section D: Other Advertisements**				
**S. No.**	**Questions**	**Responses/ Codes**		**Instructions**
D1	During the movie you just watched, did you come across any advertisements or messages about…<br><br>**Note: Do not consider any brand advertisements while coding**			
		**Yes**	**No**	
A	Immunization	1	2	
B	Use of alcohol	1	2	
C	Family planning	1	2	
D	HIV AIDS (including condoms)	1	2	
E	Blood donation	1	2	
F	Washing hands	1	2	
G	Road safety (including drinking and driving)	1	2	

H	Clean environment	1	2	
I	Domestic violence	1	2	
J	Child sexual harassment	1	2	
K	Child malnutrition	1	2	
L	Anti-child labour	1	2	
M	Education/ regular schooling	1	2	
N	Exclusive and complimentary breast feeding	1	2	
O	Use of toilets	1	2	
P	Menstrual hygiene	1	2	
Q	Consumer voice	1	2	
R	Disaster management	1	2	
S	Air pollution	1	2	

Appendix 6

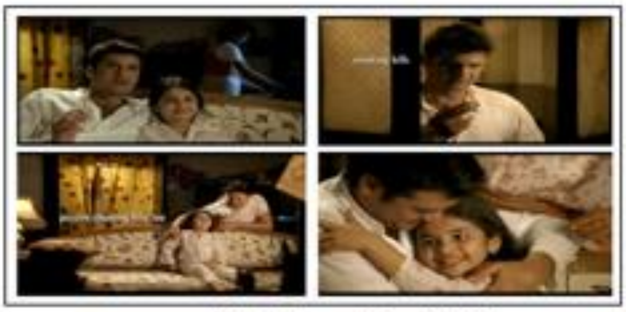

Health spot 1: Child

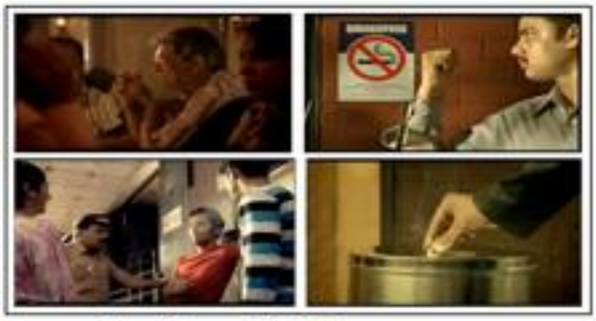

Health spot 2: Dhuan

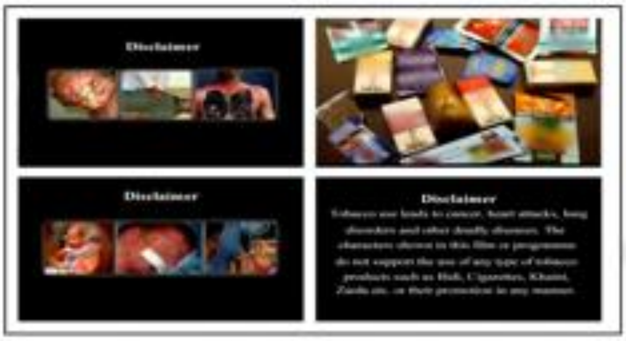

Disclaimer

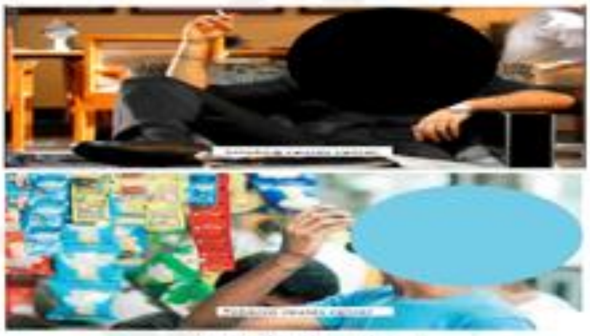

Static Message

Phases	Observations		Main Interviews	
	Start Date	End Date	Start Date	End Date
Phase 1	Feb. 3, 2015	March 4, 2015	Feb. 4, 2015	March 5, 2015
Phase 2	Feb. 16, 2015	March 23, 2015	Feb. 17, 2015	March 24, 2015
